# Temporal Information Processing and its Relation to Executive Functions in Elderly Individuals

**DOI:** 10.3389/fpsyg.2016.01599

**Published:** 2016-10-19

**Authors:** Kamila Nowak, Anna Dacewicz, Katarzyna Broczek, Malgorzata Kupisz-Urbanska, Tadeusz Galkowski, Elzbieta Szelag

**Affiliations:** ^1^Laboratory of Neuropsychology, Department of Neurophysiology, Nencki Institute of Experimental Biology of Polish Academy of SciencesWarsaw, Poland; ^2^University of Social Sciences and HumanitiesWarsaw, Poland; ^3^Department of Geriatrics, Medical University of WarsawWarsaw, Poland

**Keywords:** normal aging, temporal information processing, executive functions, executive planning, Tower of London, temporal-order judgement

## Abstract

Normal aging triggers deterioration in cognitive functions. Evidence has shown that these age-related changes concern also executive functions (EF) as well as temporal information processing (TIP) in a millisecond range. A considerable amount of literature data has indicated that each of these two functions sets a frame for our mental activity and may be considered in terms of embodied cognition due to advanced age. The present study addresses the question whether in elderly subjects the efficiency of TIP is related to individual differences in EF. The study involved 53 normal healthy participants aged from 65 to 78. In these subjects TIP was assessed by sequencing abilities measured with temporal-order threshold (TOT). It is defined as the minimum time gap separating two auditory stimuli presented in rapid succession which is necessary for a subject to report correctly their temporal order, thus the relation ‘before-after.’ The EF were assessed with regard to the efficiency of the executive planning measured with the Tower of London-Drexel University (TOL^DX^) which has become a well-known EF task. Using Spearman’s rank correlations we observed two main results. Firstly, the indices of the TOL^DX^ indicated a coherent construct reflecting the effectiveness of executive planning in the elderly. Initiation time seemed dissociated from these coherent indices, which suggested a specific strategy of mental planning in the elderly based on on-line planning rather than on preplanning. Secondly, TOT was significantly correlated with the indices of TOL^DX^. Although some of these correlations were modified by subject’s age, the correlation between TOT and the main index of TOL^DX^ (*‘Total Move Score’*) was rather age resistant. These results suggest that normal aging may be characterized by an overlapping of deteriorated TIP and deteriorated EF.

## Introduction

In recent decades societies worldwide have been facing demographic changes. A growing number of people aged 60 and older in most European countries and in the US has led to a rapid growth of research studies on cognitive functions in the elderly. Aging triggers significant decline in cognitive functioning. Although it is difficult to define exact moment when a mental activity begins to deteriorate, a robust body of experimental evidence has documented that cognitive aging is a dynamic process and refers to declined memory, new learning, attention, perception, multisensory integration, language, motor control, speed of processing, and executive functions (EF, e.g., Salthouse, 2009; Szelag et al., 2010; Setti et al., 2011a,b). The results gathered from numerous samples encompassing a wide range of ages indicated that, despite pronounced individual differences, such deterioration increases markedly after 65 years of life. The accumulated data have also indicated age-related deterioration in temporal information processing (TIP, e.g., Fitzgibbons and Gordon-Salant, 1994; Kumar and Sangamanatha, 2011; Turgeon et al., 2016). These literature data have been supported by the results of studies conducted in our laboratory (Kolodziejczyk and Szelag, 2008; Szymaszek et al., 2009). As patterning in time is one of characteristic features of human cognition (e.g., Pöppel, 1997), one may assume that TIP constitutes a neural basis for a mental activity in norm and pathology, including cognitive decline in normal healthy aging (e.g., Teixeira et al., 2013). Given the importance of TIP for our cognitive functions, the present paper focuses on the relationships between deteriorated TIP and deteriorated EF in advanced age. These relationships seem to be of great importance to understand the mechanisms of normal cognitive aging in terms of embodied cognition (Borghi and Cimatti, 2010).

### EF in Controlling Mental Activity

Executive functions are high demand meta-cognitive processes that guide the optimization of goal-oriented behaviors in unfamiliar circumstances (Etnier and Chang, 2009). Despite a long discussion among neuropsychologists about the nature and definition of EF, researchers agree, in general, that these top-down processes make it possible to plan, execute and control intentional actions. In particular, EF include planning of a sequence of processes to complete a goal, inhibition of distracting events and prepotent responses, as well as management of multiple tasks or subprocesses that make up a complex tasks performance. EF serve to control, monitor and adapt our thoughts and behaviors to changing environmental circumstances (see Diamond, 2013 for a review). EF allow to make future plans, play with ideas, as well as to change ineffective behavior, if necessary.

Numerous studies have revealed vulnerability of EF to the effects of age (see Jurado and Rosselli, 2007 for a review), similarly as that of TIP in the millisecond time range (Szymaszek et al., 2009). Starting with the framework proposed by Miyake et al. (2000), it has been emphasized that EF comprise several constructs, including distinct components of inhibition, scheduling, planning, working memory, coordination, and sequencing (Colcombe and Kramer, 2003). Among EF one can distinguish higher-order problem solving, specifically executive planning abilities, which constitute the topic of the present study.

Some researchers have emphasized that planning is one of the main constructs of EF and that it involves several interrelated but dissociable components (Colcombe and Kramer, 2003; Romine and Reynolds, 2005; Hung et al., 2013). They include identification of goal state development of sub-goal representations, anticipation of consequences, determination of requirements and integration of behaviors for achieving sub-goals (Sternberg and Ben-Zeev, 2001).

One of the tasks developed to measure this aspect of EF is Tower of London Task (TOL), the most commonly used planning-related neuropsychological tool (Chang et al., 2011). The original paradigm was invented by Shallice (1982). Since then many versions of this test have been applied in the studies of EF, however, the main goal of this task is to mentally plan a series of moves to match the configuration of beads presented by the examiner. The performance involves planning of sequential moves that are executed, monitored and modified in accordance with an overall plan of action, problem constraints and error feedback (Lezak, 2004). The test performance correlates with the outcomes of tasks evaluating other cognitive functions, i.e., attention (Culbertson and Zillmer, 2005), working memory, flexibility, and inhibition control (Welsh et al., 1999; Huizinga et al., 2006; Luciana et al., 2009).

Planning action depends on control and effectiveness of many cognitive functions. Successful solving of TOL problems requires cooperation of working memory, decision-making, inhibitory control, mental flexibility and sustained attention (Lezak, 2004). According to the authors of the TOL version applied here (Tower of London-Drexel University, TOL^DX^), the measurement is sensitive to executive problem solving and planning, behavioral inhibition and impulsivity control, attentional allocation, cognitive flexibility, abstract/conceptual reasoning, rule-governed behavior and monitoring (Culbertson and Zillmer, 2005). Several studies have revealed that the abilities measured with TOL (or Tower of Hanoi) decreased in patients with dysfunctions resulting from frontal lobe lesions, as well as in normal healthy people in advanced age. This age-related decrease starts at the age of 60 (Zook et al., 2006), 65 (Brennan et al., 1997), or even later, in the eighth decade of life (Davis and Klebe, 2001). Some authors link declined EF with general slowdown of pace of information processing and its contribution to working memory efficiency (Fisk and Warr, 1996).

Age-related changes in EF find its support in the hypothesis of frontal lobe contributions to mental deterioration in aging, assuming that declined cognitive function may be associated with changes in the structure and functionality of frontal lobes (West, 1996). They lead, in consequence, to noticeable decline in various aspects of EF and other non-executive cognitive functions, such as attention, memory, motor control, etc. Structural changes are mostly associated with the reduction in gray and white matter volume of frontal lobes, while functional changes are reflected, e.g., in compensatory higher activity in prefrontal cortex or abnormal functional connectivity in frontal regions (for review see Grady, 2012).

To explain individual differences in age-related changes in EF one may refer to recent neurocognitive models developed to explain deficient or preserved performance in the elderly (Davis et al., 2008; see also Reuter-Lorenz and Park, 2010 and Sala-Llonch, Bartrés-Faz and Junqué, 2015 for a recent summary). For example, PASA model refers to posterior–anterior shift of activity in aging and assumes enhanced activity in anterior regions, including prefrontal cortex (e.g., Grady et al., 1994; Cabeza et al., 1997, 2004; Davis et al., 2008). On the other hand, HAROLD model (Hemispheric Asymmetry Reduction in Older Adults by Cabeza et al., 1997, 2002; Cabeza and Dennis, 2012) associates compensatory mechanisms with more bilateral activations in prefrontal cortex. However, higher activation in anterior regions in aging does not necessarily have to be accompanied by better performance. This phenomenon was pointed out in CRUNCH model (Compensation-Related Utilization of Neural Circuits Hypothesis; Reuter-Lorenz and Cappell, 2008). Accordingly, the engagement of more resources at lower task demands may result in reduced resources available in more demanding tasks (Reuter-Lorenz and Cappell, 2008; Reuter-Lorenz and Park, 2010). Finally, the STAC model (Scaffolding Theory of Aging and Cognition; Park and Reuter-Lorenz, 2009) proposes the engagement of alternative neural circuits that allow to maintain a high level of cognitive function in the elderly.

Considering this body of evidence the relations may be anticipated between age-related changes in frontal lobe and tasks that pertain to EF.

### TIP in Controlling Mental Activity

It is commonly accepted that chronological age does not correspond perfectly to mental age, as two people may be of the same age, but differ in their mental capacity (Szelag et al., 2011). A challenging problem is to identify neural processes (or mechanisms) that account for poorer EF in late adulthood. As mentioned at the very beginning, temporal dynamics of information processing creates a neural frame for many cognitive functions, including EF (Pöppel, 1997, 2009; Szelag et al., 2010). Evidence from both various clinical populations and normal sample has indicated that numerous cognitive functions are rooted in the exact temporal template which creates a neural frame for the optimal mental activity. A disordered time frame is often reported in different neurodevelopmental and neurodegenerative deficits, as well as in healthy aging (Fink et al., 2005; see Teixeira et al., 2013 for a review). For example, to create and execute mental activities effectively one has to monitor the passage of time, to react and to change our behavior in time. TIP is embedded in EF such as, for instance, planning, evaluation of previous actions and decision making.

The idea of time being inherent in human cognition is not new. It derived from philosophical ideas (e.g., James, 1890) and has since been investigated in many psychological, psychophysical, and neuroimaging studies (e.g., Pöppel, 1997; Nenadic et al., 2003; Lewandowska et al., 2010; Bedard and Barnett-Cowan, 2016). In general, TIP has been categorized into two major time scales, i.e., milli- and multisecond one. On the basis of various experimental paradigms and subject populations, data has indicated that on these two levels TIP may be influenced by various subject-related factors, among which subject’s age seems one of the most important (for the overview see Szelag et al., 2004, 2010, 2011; Bao et al., 2013; Allman et al., 2014; Matthews and Meck, 2014).

The evidence supporting TIP deteriorations resulting from cognitive aging indicates reduced temporal acuity and comes from experiments on duration discrimination, temporal generalization, temporal bisection, time estimation, gap detection, and perception of temporal order (see Allman et al., 2014; Matthews and Meck, 2014; Turgeon et al., 2016 for reviews). The latter paradigm is applied in the present study and concerns the ability to sequence incoming information, considered as a neural basis of the identification of events (Pöppel, 1994, 1997; Szelag et al., 2004). Several studies have revealed that the identification of temporal order of two acoustic stimuli is only possible when they are separated by an inter-stimulus interval of at least 20–60 ms (Hirsh and Sherrick, 1961, see also Szelag et al., 2004; Wittmann and Fink, 2004 for reviews). Nearly twice as long an interval is required in the elderly, which suggests declined temporal resolution. This supports the notion of temporally discrete information processing within some tens of milliseconds time window (see e.g., Pöppel, 1997; Wittmann, 1999; Szelag et al., 2001) and suggests the existence of an internal timing mechanism that controls our sequential information processing ability (Hirsh and Sherrick, 1961).

The most interesting result was that the sensitive indicator of declined TIP was not the subjects’ chronological age, but cognitive competencies (Fink et al., 2005; Teixeira et al., 2013). Seniors beyond 65 years with TIP declined to a lesser extent showed relatively better preserved cognitive status than those with more severely deteriorated TIP (Szelag et al., 2011).

These data may find some support in the processing-speed theory of cognitive aging by Salthouse (1996), which binds cognitive deficits commonly observed in the elderly with a slower pace of information processing. Accordingly, age-related changes contribute to the decrease in the speed and amount of mental operations that can be processed at a time. Moreover, the reduced speed of processing leads to difficulties in efficient planning, executing and completion of mental processes because of time limitations for these operations in our mental activity.

### Study Aims

The relation ‘TIP – EF’ has not been investigated comprehensively in the existing literature. The present study, therefore, offers an important new approach addressing the question whether in elderly individuals the differences in millisecond TIP are related to declined EF. Although both these functions (i.e., TIP and EF) were investigated in the previous studies separately (see above), to our knowledge no studies to date have examined the associations between age-related changes in these two domains.

Considering these limitations, the aim of the present study was twofold. Firstly, we attempted to clarify the relationships between particular indices of the EF assessed with the TOL^DX^ test in subjects beyond 65 years of life. We expected to replicate some previous findings which were focused on much wider range of ages than those tested in our study. Secondly, to understand the relation ‘TIP – EF’ we verified the correlations between the indices from the TOL^DX^ and effectiveness of TIP assessed with sequencing abilities in the millisecond time range.

## Materials and Methods

The study was approved by the local Ethical Commission at the University of Social Science and Humanities (permission no 3/II/11-12). All participants gave their written informed consent prior to the study.

### Participants

Fifty-three elderly participants (46 females/7 males), aged 65 to 78 (*M* = 69.1; *SD* = 3.4) took part in the study. They were right-handed (Edinburgh Handedness Inventory, EHI, Oldfield, 1971) and free of any neurological and psychiatric disorders or systemic diseases. The abovementioned criteria were verified during interviews and geriatric examinations performed by professional geriatricians from the Geriatric Clinic of Medical University of Warsaw. The geriatric assessment included physical examination, assessment of functional performance and evaluation of currently taken medications. Individuals with supposedly poor or unstable health conditions or those receiving medications affecting the central nervous system were excluded from the study.

All participants had a normal hearing level (Ansi, 2004) in the frequency range from 250 to 3000 Hz. Frequencies included: 250, 500, 750, 1000, 1500, 2000, and 3000 Hz and encompassed the sound frequency spectrum presented in our study. An inclusion criterion for all participants was that the difference between these frequencies, as well as between the left and right ear (for a given frequency) did not exceed 30 dB.

Prior to the experiment proper all participants completed Mini-Mental State Examination (MMSE, Folstein et al., 2001) to screen for dementia. An inclusion criterion was the score of at least 27 points on this examination. Additionally, Geriatric Depression Scale (GDS, short form; Shiekh and Yesavage, 1986) was completed to screen for depression. The candidates who scored above 5 points on this scale were excluded from the study.

Verification of all the abovementioned criteria allowed us to expect that the subjects included into our sample were in relatively good health, both physically and mentally. Thus, it may be assumed that they exhibited the level of mental functions characteristic for normal, healthy cognitive aging. The characteristics of the subjects sample are presented in **Table 1**.

**Table 1:** The characteristics of the subjects sample.

*Variable*	*N*	*Mean*	*SD*	Range
Age (years)	53	69.1	3.4	65–78
Sex (female/male)	46/7	-	-	-
Education (years)	53	15	2.7	9–21
GDS (score)	53	2	1.7	0–5
MMSE (score)	53	29.2	0.8	27–30
EHI (score)	53	89	13.1	60–100
				

*GDS, Geriatric Depression Scale (short form); MMSE, Mini-Mental State Examination; EHI, Edinburgh Handedness Inventory.*

### Experimental Procedures

Two experimental procedures were applied in this study: (1) Tower of London task and (2) Temporal-Order Judgment task. These procedures were completed by the subjects during individual sessions conducted by the experimenter.

#### Tower of London Task

**FIGURE 1:**
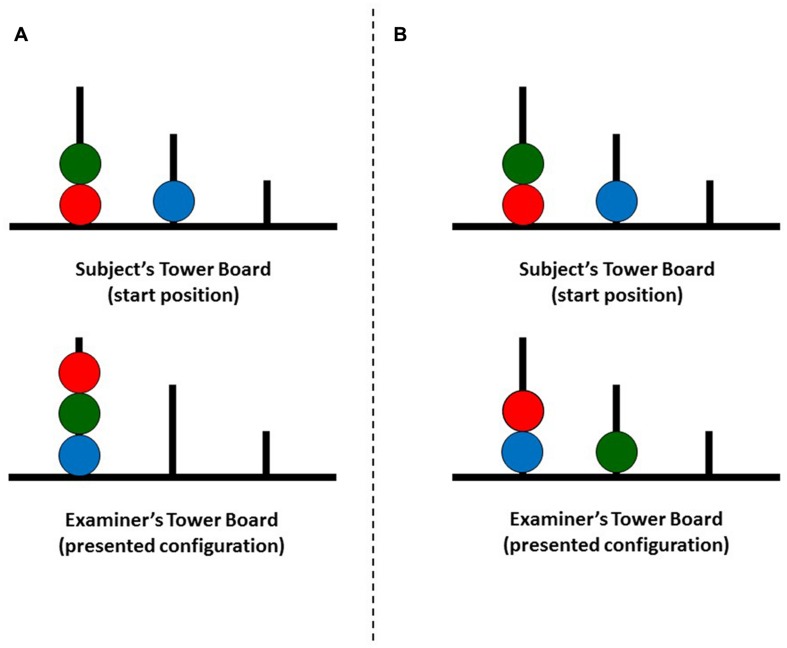
**Two examples (A,B) of Tower of London-Drexel University (TOL^DX^) problems with a minimum number of 6 moves necessary to achieve the presented configuration**.

Executive functions were assessed with Tower of London-Drexel University task: 2nd Edition (TOL^DX^, Culbertson and Zillmer, 2005)^1^. The test consisted of two identical tower boards, one for the participant and one for the examiner. Each structure contained a board with three pegs and a set of three beads (red, green, and blue). The participant was instructed to replicate configurations of beads presented on the examiner’s tower board in as limited number of moves as possible. During the execution of each trial the participant had to follow two rules: (1) it was prohibited to place more beads on a peg than it could accommodate, and (2) one bead could be moved from pegs at the time while other beads had to be kept on pegs. The examples of experimental procedure are displayed in **Figure 1**.

The task was described in a technical manual (Culbertson and Zillmer, 2005) and consisted of 10 trials of increasing difficulty, which corresponded to the minimum number of moves necessary to replicate the presented configuration. Accordingly, the entire test included two trials with the minimum of four moves, three trials with five moves, three trials with six moves, and two trials with seven moves. Prior to the task proper, the participant completed two practice trials with two moves.

The duration of each trial, including the inspection and execution of a presented target position, was measured by the experimenter. Time limit for each predetermined target position was 1 min. If this limit was exceeded, the trial was classified as time violation (see **Table 2**). The subject was not informed about any time limitation.

**Table 2:** The TOL^DX^ outcome measures grouped into three main categories reflecting cognitive processes underlying the test performance.

Category	Particular outcome measures	Definition	Associated cognitive processes
MOVE PERFORMANCE	Total Move Score (TMS)	The number of moves that exceeded the minimum number of moves necessary to replicate configurations presented by the examiner	Quality of executive planning
	Total Correct Score (TCS)	the number of trials solved in a minimum number of moves	Working memory capacity and control
TIME EFFICIENCY	Initiation Time (IT)	The time between the presentation of the configuration by the experimenter and removing the first bead from the peg by the participant	Inhibitory response processes and preparation of planning
	Execution Time (ET)	The time needed to solve each trial measured from the first to the last move by the participant	Speed or pace at which executive plans are operationalized
	Total Time (TT)	The sum of the IT and TET	Overall speed of executive planning and problem-solving speed
VIOLATION ADHERENCE	Total Time Violations (TTV)	Number of trials in which the TT exceeded 1 min	Ability to plan and execute problems solving in a specific temporal period, cognitive processing control
	Total Rule Violations (TRV)	The number of violation of required rules	Ability to govern and control executive planning according to the applicable restrictions

We analyzed seven outcome indices reported by Culbertson and Zillmer (2005). They were grouped into three main categories (Hung et al., 2013): (1) Move Performance (*‘Total Correct Score,’ ‘Total Move Score’*), (2) Time Efficiency (*‘Initiation Time,’ ‘Execution Time,’ ‘Total Time’*) and (3) Violation Adherence (*‘Total Time Violations,’ ‘Total Rule Violations’*) which are briefly summarized in **Table 2**.

#### Temporal-Order Judgment Task

**FIGURE 2:**
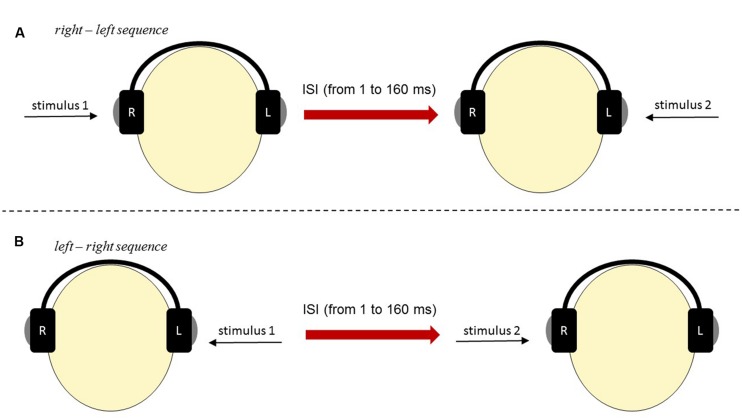
**The scheme of stimuli presentation in TOJ Task: (A) *‘right–left’* sequence and (B) *‘left– right’* sequence**.

To measure the effectiveness of auditory perception of temporal order we used two-element sequences of acoustic stimuli (for detailed description see Szymaszek et al., 2009; Oron et al., 2015). They were paired clicks (rectangular pulses) of 1 ms duration each. The stimuli were generated by a 16-bit Sound Blaster Extigy Sound Card and delivered at a comfortable listening level *via* the Sennheiser HD 205 headphones. Paired clicks were presented monaurally, i.e., one click was exposed to the left ear and the other click to the right ear. These clicks were separated by various inter-stimulus intervals (ISIs), which reflected the time gap between the offset of the first click and the onset of the second click within a pair. The participant reported the temporal order of two clicks presented in rapid sequence by pointing to one of two response cards. Two alternative cards were used: ‘*right-left*’ or ‘*left-right.’* Experimental situation is displayed in **Figure 2**. The values of ISI applied in this study varied from 1 to 200 ms, according to an adaptive maximum-likelihood-based algorithm (Treutwein, 1997; Szymaszek et al., 2009). First, using up-and-down testing ten paired stimuli were presented with constant decreasing ISIs of 160, 120, 81, 41 and 1 ms following by increasing ones of 1, 41, 81, 120 and 160 ms. On the basis of subject’s response correctness, the program estimated the probability of making a correct response to a stimulus having a given ISI according to Treutwein (1997, p. 131). Next, ISI in each trial was adjusted adaptively on the basis of correctness of the subject’s previous responses. The algorithm of the up-and-down method decreased the ISI following a correct response and increased ISI after incorrect responses. ISIs in the consecutive trials were determined by YAAP algorithm based on maximum likelihood parameter estimation. Each session was terminated when a 95% confidence was reached within ±10 steps. The criterion was threshold value within ±10 ms confidence interval at 75% probability level. Temporal-order threshold (TOT) reflected effectiveness of TIP and sequencing abilities.

An introductory session was conducted prior to the experiment proper to familiarize the participant with the task. In this session constant, relatively long ISI of 160 ms was applied in 12 consecutive trials. Once the pre-defined criterion of 10 correct responses was achieved in the last 11 successive trials, the proper experiment started.

### Statistical Analyses

First, Kolmogorov–Smirnov test was applied to verify normal distribution of the data achieved. As the results showed that more than 50% of the data was not normally distributed, non-parametric Spearman’s rank correlations were applied to investigate: (1) the performance of the subjects on TOL^DX^ task, and (2) the relationships between the outcome measures obtained on TOL^DX^ and TOJ.

## Results

The results obtained in both tasks are summarized in **Table 3**. The score of the sample tested are presented with reference to the results obtained in the previous studies (last column) for the comparable age group to that tested presently. Reference data were previously published in (1) normalization study for the TOL^DX^ (Culbertson and Zillmer, 2005; Table 4.2, p. 47) and (2) in our previous reports for the TOT (Szymaszek et al., 2009, p. 141).

**Table 3:** Descriptive statistics of results obtained in TOL^DX^ and TOJ tasks.

	Measurement	*Mean*	*SD*	Range	Reference data Mean (±SD)
	**TOL^DX^**				
*Move Performance*	TCS	4.5	2	1–9	3.3 (±1.7)
	TMS	33.2	17.5	1–75	38.8 (±15.6)
*Time Efficiency*	IT (s)	75	40.5	29–193	72 (±64.6)
	ET (s)	304.8	112.9	111–598	285.3 (±119.7)
	TT (s)	380.1	143.4	111–799	357.4 (±159.5)
*Violation Adherence*	TTV	1.7	1.4	0–5	1.0 (±1.4)
	TRV	0.9	1.4	0–5	0.7 (±1.4)
	**TOT (ms)**	92	32	32–162	80–90

*TCS, Total Correct Score; TMS, Total Move Score; IT, Initiation Time; ET, Execution Time; TT, Total Time; TTV, Total Time Violation; TRV, Total Rule Violation; TOT, Temporal-Order Threshold.*

### The Characteristic of Performance on TOL^DX^ and TOJ Tasks

As presented in **Table 3**, mean values of the particular outcome measures of the TOL^DX^ task obtained in this study were placed within one standard deviation from the results obtained in the normalization of TOL^DX^ task for the American-Canadian sample in the similar age group (60- to 80-years old, *n* = 39; Culbertson and Zillmer, 2005; Table 4.2, p. 47).

**FIGURE 3:**
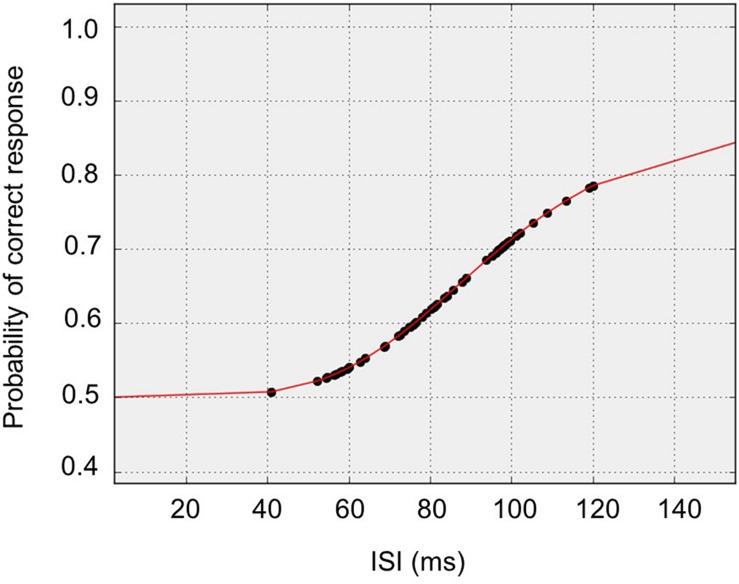
**Median subject data: typical responses and equation fit from an exemplar participant whose data was close to median responses**.

In the present study, mean TOT value was 92 ms (±32ms). This result is consistent with the data from our previous study where participants aged from 60 to 69 showed the TOT value between 80 and 90 ms (Szymaszek et al., 2009, p. 141). **Figure 3** shows typical responses and equation fit from an exemplar participant whose data were close to median responses.

On the basis of this evidence, we may assume that the subjects included into the present study were within the normal limit for both EF (assessed with TOL^DX^) and TIP (assessed with TOJ).

### The Relationships Observed in the TOL^DX^ Task

In order to better understand the relationship between EF and timing we analyzed thoroughly how the participants performed in TOL^DX^ test and conducted correlational analyses between particular indices of this test. First, we studied the correlations between indices within a given category (**Table 2**) followed by indices between categories. The results are described below and summarized in **Table 4**.

**Table 4:** Spearman’s rho correlations coefficient values (and significance levels) between TOJ and particular outcome measures of TOL^DX^.

			TCS	TMS	IT	ET	TT	TTV	TRV
	Move Performance	TCS							
		TMS	**-0.70^∗∗∗^**						
**TOL**^DX^	Time Efficiency	IT	**0.29^∗^**	-0.23					
		ET	**-0.49^∗∗∗^**	**0.76^∗∗∗^**	0.06				
		TT	**-0.33^∗^**	**0.65^∗∗∗^**	**0.29^∗^**	**0.94^∗∗∗^**			
	Violation Adherence	TTV	**-0.32^∗^**	**0.61^∗∗∗^**	0.25	**0.89^∗∗∗^**	**0.89^∗∗∗^**		
		TRV	**-0.37^∗∗^**	**0.37^∗∗^**	**-0.33^∗^**	**0.41^∗∗^**	0.25	**0.28^∗^**	
**TOJ**		TOT	**-0.33^∗^**	**0.46^∗∗∗^**	0.06	**0.34^∗^**	**0.28^∗^**	**0.33^∗^**	0.18
**TOJ**	(age controlled)	TOT	-0.20	**0.35^∗^**	0.04	0.19	0.14	0.18	0.03

*TOT, Temporal-Order Threshold; TCS, Total Correct Score; TMS, Total Move Score; IT, Initiation Time; ET, Execution Time; TT, Total Time; TTV, Total Time Violation; TRV, Total Rule Violation. Correlation coefficients that reached significance are displayed in bold with the following levels of significance: ^∗^p < 0.05, ^∗∗^p < 0.01, ^∗∗∗^p < 0.001. Additional explanations: the correlations between indices within a given category are marked with dark gray fields*.

The indices from the category Move Performance (‘*Total Correct Score’* and ‘*Total Move Score*’) displayed a strong^2^ negative correlation with each other (*r*_s_ = -0.70; *p* < 0.001). Additionally, *‘Total Correct Score’* showed significant correlations with all other measures from TOL^DX^, such as: a moderate significant relation to *‘Execution Time’* (*r*_s_ = -0.49; *p* < 0.001), weak but significant correlations with *‘Initiation Time’* (*r*_s_ = 0.29; *p* < 0.04), *‘Total Time’* (*r*_s_ = -0.33; *p* < 0.02), *‘Total Rule Violations’* (*r*_s_ = -0.37; *p* < 0.01) and *‘Total Time Violations’* (*r*_s_ = -0.32; *p* < 0.05). On the other hand, *‘Total Move Score’* strongly correlated with ‘*Total Time’* (*r*_s_ = 0.65; *p* < 0.001), *‘Execution Time’* (*r*_s_ = 0.76; *p* < 0.001) and *‘Total Time Violations’* (*r*_s_ = 0.61; *p* < 0.001). We also found a weak positive correlation between *‘Total Move Score’* and *‘Total Rule Violations’* (*r*_s_ = 0.37; *p* < 0.01).

To sum up, a greater number of problems solved correctly was accompanied by fewer redundant moves, shorter total duration of test performance, shorter initiation time, shorter time of each problem solving, and fewer rule violations. On the other hand, a smaller number of redundant moves was accompanied by shorter duration of test performance, shorter time of each problem solving and fewer time- and rule- violations.

Considering the outcome measures from the category Time Efficiency, we found a very strong positive correlation between *‘Total Time’* and ‘*Execution Time’* (*r*_s_ = 0.94; *p* < 0.001), as well as a weak positive correlation between ‘*Total Time’* and *‘Initiation Time’* (*r*_s_ = 0.29; *p* < 0.04). No significant relationship was found between *‘Execution Time’* and *‘Initiation Time.’* The correlations between categories indicate that the indices of Time Efficiency correlated with those from the category Violation Adherence. Both *‘Execution Time’* and *‘Total Time’* showed strong positive correlations with *‘Total Time Violations’* (the correlation coefficient was *r*_s_ = 0.89; *p* < 0.001 for both these indices). Additionally, *‘Execution Time’* showed a positive, moderate correlation with *‘Total Rule Violation’* (*r*_s_ = 0.41; *p* < 0.002). We found also a weak to moderate negative correlation between *‘Initiation Time’* and *‘Total Rule Violations’* (*r*_s_ = -0.33; *p* < 0.02).

**FIGURE 4:**
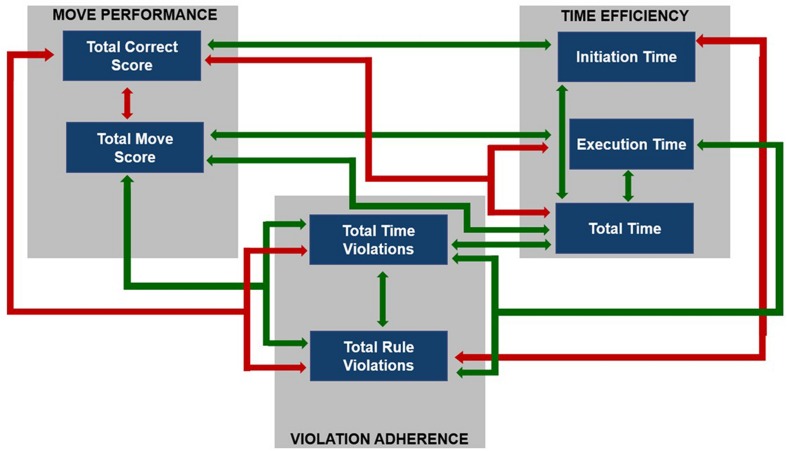
**The summary of intercorrelations between TOL^DX^ outcome measures.** Arrows indicate significant correlations: green arrows reflect positive correlations and red arrows -negative ones.

To sum up, shorter total duration of test performance (reflected by ‘*Total Time’*) was related to both shorter duration of each problem solving (strong relationship) and initiation time (weak but significant relationship). Additionally, longer time spent on solving each test problem was associated with a greater number of time- and rule-violations committed. The longer time spent before beginning of the task solving (reflected by *‘Initiation Time’*), the fewer rule violations were committed. The particular correlation coefficients and significance levels are given in **Table 4** and summarized in **Figure 4**.

### The Relationship between the Performance on TOJ and TOL^DX^

The analysis of the relationships between the performance on TOJ and TOL^DX^ tasks revealed five significant correlations between auditory TOT and particular TOL^DX^ indices (**Table 4**). Two moderate correlations between TOT and indices from Move Performance category of TOL^DX^ were shown, i.e., negative correlation with *‘Total Correct Score’* (*r*_s_ = -0.33; *p* < 0.015) and a positive one with *‘Total Move Score’* (*r*_s_ = 0.46; *p* < 0.001). In other words, lower TOT values (better performance) corresponded to a greater number of tasks solved in the minimum number of moves, as well as to a smaller number of redundant moves.

**FIGURE 5:**
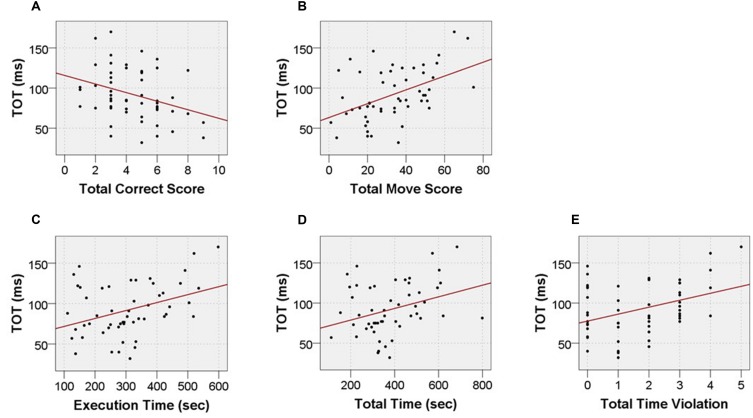
**Scatter data illustrating significant correlations between TOT values and outcome measures from TOL^DX^ test: (A) ‘*Total Correct Score,’* (B) ‘*Total Move Score,’* (C) *‘Execution Time,’* (D) *‘Total Time,’* (E) ‘*Total Time Violations***.’

**FIGURE 6:**
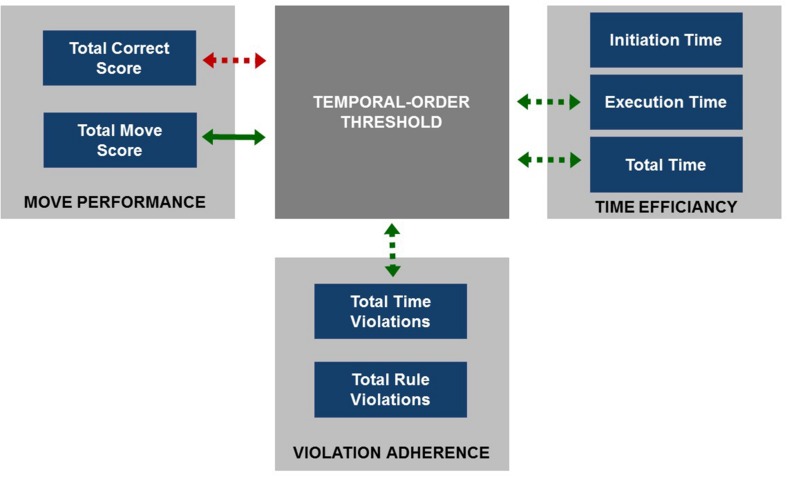
**The summary of relationships between TOT (dark gray square) and particular outcome measures of TOL^DX^ grouped into three categories (light gray squares).** Green arrows indicate significant positive correlations and the red one the negative correlation between TOT and TOL^DX^ outcome measures. The correlation significant after controlling for *‘age’* is specified with solid arrow whereas those non-significant with dashed ones.

Moreover, we pointed to two weak to moderate positive correlations between TOT and indices from Time Efficiency category of TOL^DX^, i.e., *‘Total Time’* (*r*_s_ = 0.28; *p* < 0.041) and *‘Execution Time’* (*r*_s_ = 0.34; *p* < 0.013). It means that lower TOT values corresponded to shorter both total TOL^DX^ test performance and each problem solving duration. Furthermore, for the category Violation Adherence we revealed a moderate positive correlation between TOT and *‘Total Time Violations’* (*r*_s_ = 0.33; *p* < 0.017). Lower TOT (better performance) was accompanied by a smaller number of time violations. These relationship are displayed in the **Figures 5** and **6** and summarized in the **Table 4**.

To test whether the relationship between TOT and TOL^DX^ indices may be mediated by age, we used partial correlation analysis (**Table 4**). Controlling for *‘age’* partial correlations between TOT and four TOL^DX^ indices (i.e., *‘Total Correct Score,’ ‘Execution Time,’ ‘Total Time’* and *‘Total Time Violation’*) turned out non-significant. The relationship between TOT and *‘Total Move Score’* remained significant but slightly diminished (*r*_s_ = 0.35; *p* < 0.05). These findings suggest that *‘Total Move Score’* in the lifespan tested here seemed rather resistant to subjects’ age, but more depended on TIP resources. The other indices, i.e., *‘Total Correct Score,’ ‘Execution Time,’ ‘Total Time,’* and *‘Total Time Violation’* were more age-dependent.

## Discussion

The study aimed at testing the relationships between particular indices of EF assessed with TOL^DX^ test in subjects beyond 65 years of life, as well as to understand the relation ‘TIP – EF’.

### Summary of Results

To sum up, the data obtained in both TOL^DX^ and TOJ tasks seem to be within a typical range for elderly people. In TOL^DX^ task the analyses have revealed that the values of the indices obtained are consistent, in general, with the reference data available (Culbertson and Zillmer, 2005) and correlate significantly both within and between categories (**Figure 4**; **Table 4**). A smaller number of redundant moves was related to a greater number of correctly solved test problems. Better performance (assessed with two indices from the category Move Performance) was related to shorter total test duration, shorter time of solving each of the problems, as well as less frequent violations of rules and time limits. Although any relation between initiation time and the number of redundant moves was missing, the subjects who spent more time before starting the task execution solved more test problems correctly (a weak correlation).

With regard to the relationships between the performance on TOL^DX^ and TOJ tasks, we have found that better performance on TOJ (reflected in lower values of auditory TOT) was associated with better performance on TOL^DX^ (**Figures 5** and **6**; **Table 4**) Specifically, subjects characterized by lower TOT made fewer redundant moves on TOL^DX^ and solved more problems correctly. These subjects completed the whole TOL^DX^ test faster and executed each of test problems quicker, which was associated with less frequent time violations. It should be stressed that no significant relations between the performance on TOJ and initiation time, as well as the number of rule violations, was found. After controlling for *‘age,’* the correlation between TOT and *‘Total Move Score’* was confirmed, whereas correlations between TOT and other TOL^DX^ indices were non-significant.

### Executive Planning in the Elderly

Before discussing the importance of ‘TIP – EF’ associations in the elderly, we explain the interrelations between particular indices obtained in TOL^DX^ test. Although many existing studies have shown that executive planning measured with different versions of TOL tests decreases with age (e.g., Bugg et al., 2006), to our best knowledge, none of these studies concentrated on complex relations among different performance measures on TOL tests in the elderly. Understanding of the coherence of interrelations between parameters measured with this test may be helpful in our interpretation of the associations between TIP and EF (see below).

The pattern of performance in TOL^DX^ test observed in our study was, in general, similar to that reported in the previous literature in younger groups, but we found some important differences. We confirmed the correlations between the indices within the category Move Performance (i.e., *‘Total Correct Score’* and ‘*Total Move Score’*), as well as proved their associations with those from the category Violation Adherence (**Figure 4**), indicating the relations between these two categories in the elderly. Such associations seem important, however, were not analyzed thoroughly in published reports in younger adults. The nature of these relations seems reasonable. The greater number of correctly solved problems is obviously accompanied by fewer redundant moves and fewer violations (of both time and rules).

Furthermore, we confirmed previously reported associations in younger adults between indices from categories Move Performance (i.e., ‘*Total Correct Score,’ ‘Total Move Score’*) and Time Efficiency (i.e., ‘*Execution Time,’* ‘*Total Time’*), **Figure 4**, **Table 4**. As more moves require more time, both *‘Execution Time’* and *‘Total Time’* may be considered as derivatives of motor acts executed during test solving.

These results confirm that all parameters mentioned from these three categories constitute a coherent construct of executive planning and point to some similarities in its specificity in the elderly and in younger groups, tested in previous studies. Beside these similarities, we found some important differences in the strategy of solving the TOL^DX^ task in the elderly beyond 65 years, as compared to younger groups reported in the literature. These dissimilarities concern the relations of the parameter *‘Initiation Time’* with the other performance measures.

The previous data obtained in healthy young adults (Hung et al., 2013), college students (Culbertson and Zillmer, 2005, p. 55), or adults aged from 19 to 80 years of life but considered as one subjects pool (Culbertson and Zillmer, 2005) pointed to moderate or even strong interrelations between initiation time and move efficiency (reflected in both ‘*Total Move Score’* and *‘Total Correct Score’*). In contrast, our results showed no significant correlations between ‘*Initiation Time’* and ‘*Total Move Score,’* accompanied by only a weak correlation with *‘Total Correct Score*’ (**Figure 4**; **Table 4**). It seems, thus, that *‘Initiation Time’* was only weakly associated with move efficiency indices, reflecting quality of executive planning, as well as working memory capacity and control (**Table 2**).

These results suggest that in the elderly the effectiveness of executive planning cannot be directly attributed to the time period before executing motor acts. The existing literature data on the role of planning on this test in younger adults have shown contradictory results. For example, Unterrainer et al. (2003) indicated that the participants who were instructed to make full mental plans before beginning to execute movements (preplanning) solved significantly more problems than the subjects starting the task immediately with task-related movements (on-line). On the contrary, Phillips et al. (2001) showed that the time spent on such preplanning did not necessarily contribute to better performance on TOL task, even if the instruction on taking time to plan actions was explicitly given. They evidenced that better performance in TOL task may not necessarily depend on the time spent on preplanning, but on the ability to plan on-line, i.e., to monitor and plan movements during the execution of particular test problems. Such strategy was also suggested in the earlier work of Gilhooly et al. (1999), who stated that neither young nor elderly participants made a full plan before starting the execution of trials of the TOL task. However, these authors only relied on group comparisons without any correlation analysis between the test indices.

In our opinion, these literature controversies on the planning strategy resulted from uncontrolled contribution of other mental processes incorporated in planning abilities. They involve diminished inhibitory control of actions, mental processing speed and impulsivity of a participant (Culbertson and Zillmer, 2005; Luciana et al., 2009). Many previous studies reported a decline of abovementioned cognitive skills (e.g., Salthouse, 1996; Christ et al., 2001), as well as age-related differences in cognitive styles, i.e., reflection-impulsivity (Coyne et al., 1978).

The hypothesis on the contribution of other mental processes to the planning strategy may be supported by a negative correlation between ‘*Initiation Time’* and *‘Total Rule Violation’* found in our study (**Figure 4**; **Table 4**). It suggested that the participants who spent less time before executing movements committed more rule violations, which reflected difficulties in both postponing of action execution and working under constrains imposed on them by the test instructions.

To sum up, our results indicate that in the elderly, despite a coherent construct of some indices of executive planning, ‘*Initiation Time*’ seems rather dissociated from these indices and weakly associated with the overall performance on TOL^DX^. Therefore, we argue that the elderly may use a different strategy of planning than the younger subjects, i.e., they rely not so much on the preplanning, but rather on the on-line planning during solving particular TOL^DX^ test problems.

### Associations between TIP and Executive Planning in the Elderly

Assuming the importance of TIP for our cognitive function (see Introduction), the primary goal of the present study was to test the relation ‘TIP – EF’ in normal aging in order to understand mental processes characteristic of this period of life. The results reported here confirmed declined temporal acuity in normal healthy elderly (e.g., Bedard and Barnett-Cowan, 2016; Turgeon et al., 2016) and fully matched the data reported previously in our laboratory, when the same experimental procedure as that used in the present study was employed (Szymaszek et al., 2009; Szelag et al., 2011). We also confirmed pronounced heterogeneity in the efficiency of TIP in the subject sample tested here, reflecting individual differences (Matthews and Meck, 2014).

The relevance of the present study is a novel observation that in the elderly such subject-related variability in TIP (assessed with auditory TOT) was associated with the efficiency of executive planning (**Figures 5** and **6**; **Table 4**). However, the results of partial correlation analysis (**Table 4**) indicated that the relation ‘TIP – EF’ was modified by subject’s age in the lifespan tested here (from 65 to 78 years of life, see **Table 1**). The relation between TIP and *‘Total Move Score,’* considered as the main index of TOL^DX^ reflecting the level of executive planning quality (**Table 2**), was resistant to age-related influences, but strongly associated with TIP. Thus, timing seems to be much more related to executive planning than age in the subjects pool studied here. The persistence of the correlation between TIP and *‘Total Move Score*’ in partial correlations controlling for age supports the hypothesis on an important role of TIP for numerous mental functions, including higher-order meta-cognitive processes, such as executive planning investigated in our study. It may be assumed, therefore, that the performance on TOL^DX^ reflected by ‘*Total Move Score*’ – the main index of this test – is rooted in a defined millisecond template, creating a neural base for executive planning abilities. Our results suggest that TIP in a millisecond range is related to these top-down processes engaged in controlling and monitoring our behavior during adaptation to changing environmental conditions. Proper sequencing and maintenance of information provide matrices for building up our sensations, identification of events and ordering them chronologically. Such ability remains the major prerequisite for proper functioning of higher cognitive functions, i.e., EF. It may be suggested, therefore, that the performance on TOL^DX^ is rooted in a defined millisecond template, creating a neural base for executive planning abilities.

On the other hand, in partial correlation analysis controlling for age the relation between TOT and other TOL^DX^ indices, i.e., ‘*Total Correct Score*’ from the category Move Performance, moreover, *‘Executive Time’ and ‘Total Time’* from Time Efficiency, and ‘*Total Time Violations’* from Violation Adherence (**Table 2**) did not correlate with TIP and were rather age-related (**Figure 6**; **Table 4**). Thus, the more advanced age of participants, the poorer performance on these indices was indicated. It may result from declined concomitant functions, like working memory, behavioral control and functioning dynamics, which decline progressively after the age of 65. The lack of correlations between these indices and TIP (after controlling for age) may also, at least partly, result from the instructions given to participants that did not mention any time limitation. It seems very plausible, therefore, that participants did not perform the task at their maximum tempo. It also seems that the indices from Time Efficiency category, i.e., ‘*Total Time’* and ‘*Executive Time’* may be prone to such physiological factors as slowing of movement in aging, which are not directly related to effectiveness of mental planning but much more to subject’s age.

Referring to the specific planning strategy in the elderly (discussed above) based more on on-line planning than on preplanning and reflected in the dissociation of *‘Initiation Time’* from the other TOL^DX^ indices, we should stress that no significant correlation between the effectiveness of TIP and *‘Initiation Time’* was proven (**Figure 6**; **Table 4**). The lack of such associations may indicate that preplanning (reflected in ‘*Initiation Time,’* **Table 2**) is not so much related to timing, but rather to other processes, among which impulsivity and inhibitory control should be taken into consideration.

The final question concerns potential mechanisms or processes underlying the relationship ‘TIP – EF’ observed in our study. Despite pronounced individual differences, experimental data have indicated that the perception of succession is controlled by the internal timing mechanism, operating in some tens of millisecond time window implemented probably in neuronal gamma band oscillations with a periodicity of ca. 40 Hz (VanRullen and Koch, 2003; Oron et al., 2015) Accordingly, one oscillation period has ca. 25 ms duration. Referring to Pöppel (2009), the relation ‘before-after’ can be properly identified if two stimuli occur at least in two successive oscillatory periods. There is strong evidence that spontaneous (or stimulus triggered) gamma band oscillations, corresponding in duration to TOT, play an important role in human cognition (VanRullen and Koch, 2003).

The relation ‘TIP – EF’ on some tens of milliseconds level may suggest common neural mechanisms underlying these two mental functions. They create a temporal frame for both our auditory perception of temporal order of incoming events and EF, moreover, for other non-executive cognitive functions, among which language reception was indicated in previous reports (Fink et al., 2006; Oron et al., 2015). It may be hypothesized that the relations ‘TIP – EF’ cannot be restricted selectively to the auditory modality which was tested here. At this point one may refer to classical reports by Efron (1963) and Swisher and Hirsh (1972), who indicated that deficient TIP in aphasic patients was not related selectively to auditory processing but concerned also vision and touch. A similar ‘temporal binding window’ for audition and vision was also indicated in our previous study (Kanabus et al., 2002). It may suggest the common timing mechanism underlying event ordering which creates a frame for multimodal processing. It may be suspected that this temporal mechanism operates on a very fundamental level, regardless of the specificity of the task, i.e., it seems active in auditory perception of temporal order (reflected in TOT without any motor component) or planning and execution of moves (reflected in solving TOL^DX^ problems with a motor component). To understand the potential neural source of these very fundamental processes by means of creating the common neural operational frame on which various mental activities are embedded, one could refer to the neuronal oscillatory activity. Accordingly, expected slower gamma oscillations in advanced age might result in deficient mechanisms operating in some tens of milliseconds time window, less efficient TIP (higher TOT values) and declined executive planning, in which such timing is implemented.

Additional support for strong associations between TIP and EF comes from neuroimaging data. Our previous studies on neuroanatomical representation of TOJ in a millisecond range showed dynamic changes in neural activity depending on task difficulty (Lewandowska et al., 2010). Decreased TOJ difficulty was accompanied by increased activity in bilateral medial frontal gyri, which constitute a part of prefrontal cortex. As mentioned in Introduction, this region is involved in EF. Referring to Bayesian models (Turgeon et al., 2016), such neural network may be related to a central timing mechanism supported by the processes of neurotransmission on the synaptic level, specifically by dopamine-glutamate interactions in cortico-striatal circuits (Turgeon et al., 2016). From this perspective, normal aging is associated with possible reduction in accuracy and precision in dopaminergic functions resulting in age-related decline in TIP and EF. The overlap of neuroanatomical activity in TIP and EF may support a common activation network in which millisecond timing constitutes an essential component of EF governing human cognition.

## Conclusion

The results of the present study could shed a new light on our understanding of neural mechanisms underlying human mental activity and allow some generalizations on the taxonomy of functions in neuropsychology.

On the basis of the taxonomy proposed by Pöppel (1994), which was developed in our studies (Szelag et al., 2010, 2011), two classes of cognitive functions may be distinguished. The functions of the first class, i.e., context-related or ‘WHAT’ functions refer to the mental context of our subjective experience, like conscious percepts, language utterances, new learning material, memory traces, motor acts, etc. The functions of the second class constitute ‘HOW’ functions. They provide a formal operational basis and control the logistic prerequisite of context-related ‘WHAT’ functions. Hence, one may assume that logistic-related ‘HOW’ functions create a neural frame for our mental activity into which particular cognitive non-EF are embedded.

The question arises which functions may be classified into such logistic framework. As TIP provides the crucial component of human cognition (Pöppel, 1994, 1997, 2009; Wittmann, 1999; Allman et al., 2014; Matthews and Meck, 2014), it may be classified as the major example of ‘HOW’ functions. On the basis of a huge amount of literature evidence (see Introduction), EF including executive planning may also be considered as meta-cognitive processes or logistic functions that are responsible for starting, stopping and shifting of the other context-related, non-executive cognitive functions.

Finally, we would like to argue that both TIP and EF create a logistic basis of our mental functioning. The novel outcome of the present study is that the effectiveness of these two logistic functions is intercorrelated, which may support the notion of their common neural substrates. Thus, advanced or deficient executive planning in the elderly corresponds to the effectiveness of TIP in a millisecond range assessed with auditory TOT. The supramodal cooperation between TIP and EF may constitute an example of integrative activity of the brain.

## Author Contributions

KN and AD: Subject recruitment, acquisition, analysis and interpretation of data, manuscript writing. KB and MK-U: Subject recruitment, geriatric diagnosis of the subjects qualified into the study. TG: Interpretation of data. ES: Acquisition, analysis and interpretation of data, manuscript writing, responsibility for the final version of manuscript.

## Conflict of Interest Statement

The authors declare that the research was conducted in the absence of any commercial or financial relationships that could be construed as a potential conflict of interest.

## Footnotes

1 This version was applied as it allows to analyze complex subjects’ performance on the basis of various indices (see below).

2 Effect sizes according to Cohen (1988).
